# Homelessness as structural vulnerability: Precarious citizenship and migrant health in a Nordic welfare state

**DOI:** 10.1016/j.jmh.2026.100423

**Published:** 2026-06-26

**Authors:** Frode Eick, Synnøve Økland Jahnsen

**Affiliations:** a Lovisenberg Diaconal University College, Loviseberggata 15 B, 0456 Oslo, Norway; b Institute of Health and Society, University of Oslo, Postboks 1130 Blindern, 0318, Oslo, Norway; c The Fafo Institute for Labour and Social Research, Borggata 2B, Postboks 2947 Tøyen, 0608, Oslo, Norway

**Keywords:** Homeless migrants, Healthcare, Structural vulnerability, Mental health

## Abstract

**Background:**

Housing is a key social determinant of health, yet little is known about how homelessness shapes migrants’ health in Nordic welfare states where legal status, labour market attachment and welfare entitlements are unevenly distributed. This article examines how homelessness affects the physical and mental health of migrants, and how structural barriers limit their capacity to navigate access to health care.

**Methods:**

Exploratory mixed-methods study combining registry data on shelter use in Oslo (2019–2023) and interviews with ten frontline health and social care staff at a long-term, NGO-run shelter. Analyses were informed by precarious citizenship and structural vulnerability.

**Results:**

Homeless migrants represented a heterogeneous group in terms of citizenship status and health problems. Migrants’ region of origin influenced their resident status, the uncertainty of their situation, and the duration of their stay. Homelessness interacted with migrants’ pre-existing vulnerabilities, such as precarious legal and economic status, language barriers, limited access to public services, and untreated chronic disease, to accelerate health decline. Precarious citizenship can be enacted through gendered arenas of exploitation, with consequences for safety, access to care and possibilities for recovery.

**Conclusion:**

Homelessness among migrants reflects structural vulnerability and contributes to cumulative disadvantage. Homelessness is not simply a backdrop to migrant ill-health; it is an active health risk that exacerbates disease, disrupts treatment, and limits recovery. Shelters and low-threshold services play a crucial bridging role by enabling access to treatment and rehabilitation. Addressing migrant health inequities requires rights-based approaches and integrated policies linking housing stability and health care access.

## Highlights

• Homelessness interacted with migrants’ pre-existing structural vulnerabilities.
• Precarious citizenship is central in the marginalization of homeless migrants.
• Shelters and low-threshold services play a crucial bridging role to access care.
• Humanitarian and project-based responses have severe limitations.
• Integrated and rights-based housing responses for homeless migrants are needed.

## 1 Introduction

Homeless people have long time been considered as an at-risk group where homelessness, health and access to healthcare are closely linked (Hewetson, 1975). Homelessness is widely associated with several detrimental health outcomes. Studies have found homeless people to have increased risk of non-communicable diseases such as cardiovascular diseases (Al-Shakarchi et al., 2020; Bertram et al., 2022; Korukonda et al., 2025). Homelessness is also associated with communicable diseases such as sexually transmitted infections, latent tuberculosis infection and ectoparasite infestations (Lule et al., 2020; Ly et al., 2020). During the Covid-19 pandemic homeless people had higher rates of hospitalization and mortality than the general population (Ogbonna et al., 2023). Homeless people, especially men have increased risk of mental health problems such as substance use disorder, major depression, antisocial personality disorder and schizophrenia (Barry et al., 2024; Kelly, 2005; Maestrelli et al., 2022). Homelessness is associated with premature ageing, and subgroups are associated with increased risk of frailty (Cronin et al., 2025). Studies found homeless people experience food insecurity, lack micronutrients and that many are overweight or obese (Easton et al., 2022; Huang et al., 2022). Despite the identified health inequalities, reviews have found high heterogeneity between studies, questioning the methodological quality, and suggest improved data collection in population subgroups (Aldridge et al., 2018; Fornaro et al., 2022). There is also a need of intervention trials and coordinated services to improving health services for homeless people (Constance and Lusher, 2020; Hanlon et al., 2018).

European countries have, over the last decades, encouraged and facilitated intra-European work mobility. While many benefit from this freedom of movement, a subset of mobile European Union (EU) citizens struggle to find their way out of precarity (Brunovskis et al., 2024; Misje, 2020; Seilskjær, 2023). Public and policy discourses have increasingly framed some EU migrants at the margins of the labour market not as workers, but as social problems, often categorised as “beggars” or “welfare seekers” rather than as rights-bearing labour migrants (Holte and Misje, 2024). This categorisation resonates with broader processes of undeserving and precarious citizenship within EU welfare states (Lafleur and Mescoli, 2018). In addition, there has been an increase of migrants to Europe who seek protection and to make a decent living (Rechel et al., 2013). Migrants experiencing homelessness represent a diverse group, with variation in migration histories, legal statuses, labour market participation and health profiles (Pouille et al., 2024; Seilskjær, 2023; Tomkow, 2020). Nevertheless, their daily realities share structural characteristics: unstable or exploitative employment, limited financial security, and exclusion from basic welfare entitlements (Mburu et al., 2013). Work migrants without permanent resident permit may end up as homeless if they get in a conflict with an exploitative employer (Brunovskis et al., 2024; Thorbjornsen, 2017). The relationship between migration status, health and vulnerability for exploitation and forced labour is most acute for undocumented migrants, who often find themselves in informality/irregularity or legal grey zones (Chávez et al., 2024; Yang et al., 2023).

Although the extensive literature on the connection between homelessness, health and access to health care, the literature on homeless migrants’ health in Europe is scarce. They are seldom included in surveys or register studies, or ethnicity and migration background may not be identified in studies. As other migrants their health may be connected to migration phases and the social determinants of their health trajectory. Marginalized ethnic groups such as Roma peoples with specific challenges may also be homeless migrants outside their country of origin (Ravnbøl, 2017). Homeless migrants in France and Italy had high prevalence of respiratory symptoms (De Maio et al., 2014; Ly et al., 2019). A study from Germany found that homeless migrants had particularly negative health outcomes (Bertram et al., 2022). In Italy homeless migrants revisited healthcare more frequently than Italian-born homeless (Silvestrini et al., 2016). In contrast, a Spanish study found that being born in Spain was associated with increased mortality risk among homeless people (Calvo et al., 2025). Homeless migrants may have mental problems due to earlier experiences as in Paris Region where homeless migrant mothers had high levels of Post Traumatic Stress Disorder (Roze et al., 2020). In northern France aid-workers reported uncertainty, disempowerment and restricted services as negatively affecting the health of migrants in camps (Pursch et al., 2020).

Homelessness disrupts treatment pathways and contributes to discontinuity of care. In addition, stigma is found to be a main barrier for access to healthcare among people experiencing homelessness (Price et al., 2025; Reilly et al., 2022). Several reinforcing factors including homelessness have been identified functioning as barriers for health care among destitute migrants and marginalized groups (Li et al., 2024; O'Donnell et al., 2016; Pouille et al., 2024). Primary and specialized care services who encounter homeless migrants may provide help though rights may be restricted. However, the combination of homelessness and mobility complicates efforts to assess health needs, clarify legal entitlements, and build trusting clinical relationships (Kaur et al., 2021). Studies have found challenges such as upholding continuity in providing care to migrants in precarious situations (Eick et al., 2022; Jensen et al., 2011; Kvamme and Voldner, 2021; Rast et al., 2025; Temesvary et al., 2025; Voldner et al., 2023). However, the access to welfare benefits is not only defined by legal status but also by various factors within the working EU migrant population (Czech-Havnerås and Schweyher, 2024; Lafleur and Mescoli, 2018; Misje, 2020; Schweyher, 2023).

Stable housing as a fundamental right regardless of resident status are stated in several international legal frameworks (Mylona et al., 2025). We use the conceptualization of undeserving and precarious citizenship to understand the relation between homelessness, health and migration (Lafleur and Mescoli, 2018). Lafleur & Mescoli argue that welfare policies are since the expansion of EU in 2004 increasingly used to limit mobility of poor EU migrants. This filtering of undesired EU migrant leads to a growing preciousness among migrants. The concept of structural vulnerability emphasises how political, economic and social structures create specific vulnerabilities in migrants’ accessing healthcare and their health (Carruth et al., 2021). Together, these two conceptual perspectives orient the analysis at distinct but interrelated levels. Precarious citizenship captures how welfare regimes differentiate between categories of residents, creating graduated forms of inclusion and exclusion at the legal-administrative level. Structural vulnerability directs attention to how political, economic and administrative structures constrain migrants' access to healthcare, welfare and housing at the institutional and systemic level, and how these constraints become inscribed in individual health trajectories over time.

Recognition of migrants’ distress is found to be important establishing a trusting relationship with migrants in stressful living conditions (Andersson and Punzi, 2024; Barkensjö et al., 2018). Healthcare provider should not only be aware of structural vulnerability as a concept but operationalize it through systematic detection and influence the provision of healthcare (Bourgois et al., 2017). Outreach services, close collaboration and shelters staffed with healthcare professionals are proposed as one of the solutions to increase access to health care for homeless persons (eClinicalMedicine, 2023; Johnson et al., 2025). To facilitate customized services, we need to know more about homeless migrants and how their healthcare needs are shaped. In the present study, we draw on both precarious citizenship and structural vulnerability to examine how homelessness operates across these levels – as a condition shaped by legal status and welfare entitlements, as a relational experience of dependency and exploitation, and as a process through which health deterioration accumulates in the body. To understand the interconnection between migration, health and homelessness better this study aims to investigate the experiences from a long-term shelter aimed at homeless migrants with a health problem.

## 2 Methods

### 2.1 Design

We adopted an exploratory mixed‑methods design in which registry‑based quantitative data on long‑term shelter stays among migrants were analysed alongside qualitative interviews with frontline workers. The quantitative component provided a descriptive overview of the scale and temporal patterning of homelessness among migrants, while the qualitative component illuminated how migration status, welfare eligibility and service configurations shaped structural vulnerability in everyday practice.

### 2.2 Setting

Norway is a Northern European country with a generous welfare state for permanent residents with a public insurance scheme (Saunes et al., 2020). Those who are not considered as permanent resident have restricted rights and access to healthcare services regulated through a circular (Ministry of Health and Care Services, 2011).

Oslo is the capital of Norway with 717 710 registered inhabitants in 2023 (Statistics Norway, 2025). Night shelters with limited capacity have been established in the biggest cities by non-governmental organisations (NGO) since 2013 and are funded by the Ministry of Justice and Public Security.

### 2.3 Long-term shelter

An NGO established a long-term shelter for homeless migrants in Oslo which operated between 2019–2023, with money from a national charity campaign. The aim of the long-term shelter was to better provide adequate care, enabling rights and legal advice to homeless migrants. The shelter was first staffed daytime with a social worker, a nurse and a lawyer which later changed to two social workers and one psychiatric nurse. The long-term shelter had 11 rooms in a three-floor house with one shared kitchen in each floor and with a discrete address. The initial duration of stay was set to one to three weeks.

### 2.4 Participants and recruitment

We used a combination of purposive and snowball sampling. The shelter operated with a small, stable team, and our aim was to cover the full range of professional perspectives within this bounded informant environment rather than to pursue thematic saturation in the conventional sense. We started interviewing those who had worked at the long-term shelter and with selected persons from low threshold facilities who had referred migrants to the shelter. The last four participants were proposed by earlier participants. In total, the interviews captured the perspectives of all core staff involved in the shelter's operation as well as key referring partners.

### 2.5 Data collection

Data was collected between May to July 2025. We collected 5 documents including one registry of users at the shelter. In addition, we did seven semi-structured qualitative interviews and one focus group discussion with a total of ten health and social workers. Interviews were conducted at participants’ workplace by the first author alone or by the two authors. Interviews were directly transferred to Nettskjema. The interviews were automatically transcribed through Autotext a speech-to-text transcription service integrated in Nettskjema, and then checked and finalized by a research assistant.

### 2.6 Descriptive statistical analysis

We used R software program for statistical analysis. Descriptive characteristics of homeless migrants who were registered using the shelter were summarized as number (n) and percentage (%). Two levels of analysis were described by gender: Table 1 presents age and region of origin among 140 individuals, and reason for admission and median days at the stay level (n = 273 stays among 140 individuals), as a single migrant could have multiple stays with different reasons for admission. The registry of the long-term shelter contained the reason for admission as a short text noted by the staff at admission. The staff text to each admission was categorized into five categories (Table 2). The categories were developed by the first author inductively during familiarization with the registry, and later discussed with the second author.

### 2.7 Qualitative data analysis

We drew on collective qualitative analysis (Eggebø, 2020), following its five-step structure: data reduction, theme mapping, thematic grouping, outline and work plan, and writing with discussion and quality assurance. The first author wrote summaries of all interviews shortly after they were conducted. Both authors read the transcripts, and listened to all interviews. These summaries and readings formed the basis for joint sessions during autumn 2025, in which the authors mapped themes, grouped them into main categories, and arrived at three main themes corresponding to distinct analytical levels at which structural vulnerability and precarious citizenship operated in the material.

### 2.8 Ethics

This study was carried out in accordance with the World Medical Association Declaration of Helsinki. The processing of personal data in the study was approved by Norwegian Agency for Shared Services in Education and Research (Ref. 915,686). Study participants made an informed written consent before being interviewed. The registry of migrants using the long-term shelter 2019–2023 did not have identifiable information and were not considered as personal data. Ethical considerations were integral to the study design. We deliberately minimized the privacy burden by not interviewing migrants directly, while still obtaining important insight through staff interviews and anonymized historical registry data.

## 3 Results

### 3.1 Demographics

We found 140 homeless migrants using the shelter over the five-year period**.** The migrants were aged 18 to 80 years (Median 43, IQR 36–53) and 63.6% were men. The characteristics of male and female migrants using the shelter is presented in Table 1. They originated from 30 different countries where 72.8% reported to be EU citizens.

**Table 1:** Characteristics of migrants and their stays using long-time shelter in Oslo.

	Women		Men	
	n	%	n	%
	51	36.4	89	63.6
				
Age (years)				
Median	41 (IQR 33–49)		45 (IQR 39–53)	
				
Region of origin ^a^				
Europe and Central Asia	29	56,9	73	82,0
Middle East	1	2,0	7	7,9
Sub-Saharan Africa	14	27,5	8	9,0
South Asia	0	0	1	1,1
East Asia and Pacific	5	9,8	0	0
Latin America and Caribbean	2	3,9	0	0
Total	51	100.0	89	100.0
				
Number of stays				
Median	1 (IQR 1–1.5)		1 (IQR 1–2)	
				
Main category of reason for admission				
Health problem	25	29.1	67	35.8
Pre-/post hospital	16	18.6	26	13.9
Rest/tired	12	14.0	46	24.6
Social/practical clarification	29	33.7	28	15.0
Labour exploitation	2	2.3	18	9.6
Unknown	2	2.3	2	1.1
Total stays	86	100.0	187	100.0
				
Duration of stay in days				
Median	23.5 (IQR 13–43)		21 (IQR 11–36)	
		

a World region according to World Bank.

### 3.2 Stays

The long-term shelter accommodated 273 stays among the 140 migrants over the five-year period. Stays accommodated per year increased from 27 in 2019 to 106 in 2023. Migrants had from 1–10 stays each over the five-year period with a median of 1 (IQR 1–2). The median length of stay was 21 days (IQR 12–38). Women stayed median 2.5 days longer than men (Table 1).

### 3.3 Reason for admission

Five main categories of reasons based on reason for admission text noted by the staff in the registry of the long-term shelter are presented in Table 2. Homeless migrants had a variety of chronic and acute health problems related to living conditions, lifestyle and exposure to harm. They had been admitted preparing before hospital examinations and after discharge for serious conditions. Several had been admitted due to exhaustion of living on the street and some of them were drunk. Some homeless migrants were admitted for clarification of their situation and to see if they had rights to health and social services. Others were admitted because of possible victim of forced labour. The two main reasons for men were health problems and rest/tired whereas for women it was health problem and social/practical clarification (Table 1).

**Table 2:** The five main categories of reason for admission based on text noted by staff in the registry of the long-term shelter.

Reason for admission	Examples of text
Health problem	• Major health challenges, awaiting clarification on surgery • Wound in the foot after stepping on broken glass • Broken arm • Scabies • Schizophrenia and poorly regulated diabetes • Psychosis • Victim of rape
Pre-/post hospital	• Hospital appointment that required special preparations • Have an MRI examination • Had an abortion, needed supervision and a place to be • Heart attack, discharged from hospital • Been admitted to hospital with both breathing and heart problems, has asthma/COPD
Rest/tired	• Lost his home and needs a rest period • Rest, reality orientation • Tired, confused about his own situation • Tired and drunk
Social/practical clarification	• Pregnant, with two children, clarification, mapping • Was to be transported out the next day, delivered by the Police to a cafe for homeless people • Don't have a place to live, want a place at an asylum reception center • Uncertainty about rights • Challenges with social services, unclear whether he has rights
Labour exploitation	• Possible victim of forced labour • Labour exploitation • Tricked into coming to Norway for work • Work-related crime case

### 3.4 Characteristics of homeless migrants and their needs

We generated three main themes from our analyses, each corresponding to a level at which structural vulnerability and precarious citizenship operated in the empirical material - from the clinical-institutional interface, through relational and administrative dynamics, to cumulative embodied effects (Bourgois et al., 2017; Carruth et al., 2021; Lafleur and Mescoli, 2018). E*qual illness with unequal consequences* captures how the same health condition led to different trajectories depending on legal status and welfare entitlements. *Constant uncertainty, dependence* and *erosion of autonomy* illustrate how graduated rights and welfare bordering shaped migrants' dependency on informal networks and humanitarian services. *Deterioration of mental and physical health under housing instability* traces how sustained exclusion became inscribed in physical and mental health over time.

#### 3.4.1 Equal illness with unequal consequences

This theme illustrates how structural vulnerability operates at the clinical-institutional interface, where restricted rights and welfare exclusion transform the clinical significance of otherwise treatable conditions (Bourgois et al., 2017). The participants reported that homeless migrants had a variety of physical and mental health problems. Some were chronically sick with for example diabetes, cardiovascular diseases or an alcohol or drug addiction before they came to Norway. For others, health deteriorated or they became acutely ill when being homeless. The health problems were closely related to their precarious situation including living conditions, mental health, exposure to assaults, lifestyle, and for some being exploited through work (Table 2). When the social worker and the psychiatric nurse were able to establish a relation to the person staying in the shelter, they often found the person’s situation to be considerably more complex than the initial reason for admission suggested.

Being homeless meant that existing health problems could aggravate and became more difficult to treat. It was more difficult for doctors to stabilize the level of blood glucose when the patient with chronic conditions such as diabetes were homeless and had inconsistent access to food. Psychologists found it unsatisfactory to treat mental problems among patients who were homeless and focused on sustain life hour to hour. And nurses found it hard to provide good wound care when homeless patients lived in unhygienic conditions. Like one of the staff reported:

*“If you have athlete's foot, you won't get rid of it if you sleep outside.”* – Participant 5

From the other side, the long-term shelter gave the opportunity to provide better quality of health care such as follow up of medication for tuberculosis and other chronic conditions. One of the staff reported that stable housing had direct impact on the quality of healthcare:

*“But what is very important, which we probably never had the chance to do before, is that these are the ones that we needed to stabilize on medication, get an overview of how they took their medication, whether they took it, and things like that. Because it is very important treatment.”* – Participant 3

Without this kind of close follow up treatment, the staff believed the homeless migrants would end up with a hospital admission eventually. The long-term shelter provided therefore an opportunity for preventive healthcare and to talk about issues like how to take medication, provide health information and to prepare for examinations or treatment at the hospital. Some of the homeless migrants got several examinations over time for severe illness that would not be possible when living in the street.

Dependent on their resident status, the homeless migrants had restricted rights to various levels of primary and specialized healthcare. This meant for some that they had access to parts of the public healthcare system, but not all. Preventive care, chronic disease management, and rehabilitation were largely inaccessible. A major mechanism through which homelessness worsened health was the absence of a stable environment for post-treatment recovery. Participants described homeless migrants being discharged directly to the street after major medical procedures, with no possibility of rest, wound care or follow-up. The staff reported that they admitted people who had undergone surgery for example two days after a heart attack, brain tumour and after surgical termination of pregnancy that had been discharged to the street. Some had been discharged in a wheelchair or with a walker, others with dizziness, headache and who were sensitive to sound and light after surgery with the risk of delayed rehabilitation and complications. Some of the staff were shocked over what was regarded as completed treatment from the hospitals.

*“I remember one of the first people I met – a woman who had undergone surgery for an orbital fracture. She had undergone surgery to insert a plate behind her eye. She was treated in hospital, operated on – and then released straight back onto the streets. This was in the middle of winter.”* – Participant 7

This case shows how homelessness turns hospital discharge into a second health risk, with the possibility of complications or rapid deterioration.

#### 3.4.2 Constant uncertainty, dependence and erosion of autonomy

This theme captures the relational and administrative dimensions of precarious citizenship, showing how graduated welfare entitlements and legal instability structured migrants' dependency on others. Homeless migrants’ region of origin influenced their resident status, the uncertainty of their situation and duration of stay. The staff reported two main groups of users, migrants from EU and undocumented migrants from other part of the world. Most homeless migrants from EU were coming from East European countries with more a various length of connection to and shifting status in the Norwegian society.

*“Among EU citizens, it could vary greatly: from people who were here for a short time, for three months, to people who had lived in Norway for decades and had gone in and out of formal employment. Most had D numbers* (temporary ID number)*.”* – Participant 1

Homeless migrants struggled to navigate a highly complex health system due to the instability inherent in homelessness. The ability to access rights in the Norwegian system depended on both individual and structural factors such as mental health problems, precarious life situation, poverty, a rigid Norwegian system and lack of digital access to the social services. The staff reported that migrants lacked Norwegian language skills and knowledge about the Norwegian system which made it difficult for them to explain their situation and access social entitlements such as daily allowances and sick pay. For some becoming unemployed led to homelessness:

*“… for example, Polish men who had been in Norway for twelve years and paid taxes the whole time, still only had a D number* (temporary ID number) *because they never managed to register. When they then lost their jobs, they had to apply for unemployment benefits on paper* (compared to a digital application for those with a permanent ID number) *– a process that could take at least ten weeks. In the meantime, they became homeless, because they were not entitled to social assistance to pay rent while they waited for their application to be processed. They remained in the vicious circle.”* – Participant 1

Most undocumented migrants were women who did not get asylum, many with several years in Norway and some who needed support with difficult life choices as they did not have the authorization to stay in the country. One of the staff explained:

*“…were undocumented in very difficult situations: should they return to reception, try out in society, or go home? These were difficult cases, and we tried to support people in making important choices. These were often long psychosocial processes, perhaps not many in number, but very significant.”* – Participant 5

A characteristic of homeless migrants’ precarious situation was the uncertainty and transience of their lives which included a dependency on others such as NGOs and social network. The uncertainty included where to access food and where to stay, sleep, go to toilet, shower and store their belongings. The alternative places they could sleep were at night shelters, stay in reception centres for asylum seekers, in the street/sheds or privately through their social network.

*“Some people must circulate between five or six different places. It could be two weeks here, a week there, two weeks somewhere else. They have a kind of fixed cycle. It's very tiring – but still a better alternative than moving into a reception center, which many people find worse.”* – Participant 6

The homelessness and dependency on others meant that they had to navigate a constant and unpredictable situation being exposed to exploitation. Staff reported that undocumented migrant women stayed in domestic homes within their diaspora and ‘paid rent’ through performing various tasks such as cleaning, looking after the apartment during the owners’ holiday and babysitting. They reported stories of sexual abuse, unbalanced power relations and homeless migrants getting indebted to others. The staff worried when they met homeless migrants who obviously were in a personal relation to have somewhere to stay, or in families with uncertain emotional or physical capacity to care for them.

Staff reported that they admitted women who were victims of partner violence who did not get access to the designated public shelters, and they admitted men who possibly had been exploited through work but did not get access to the designated shelter for victims of human trafficking. Some of the undocumented migrant women needed a place where they felt safe from deportation. The long-term shelter represented a place where they felt protected. One of the staff commented it like this:

*“When they came to us, they were so scared that they couldn't sleep at night. They only slept when we got to work in the morning – because it was safe that we were there. It gave them security to know that no one was going to take them while we were in the house. I think it was a psychological burden – a lot of physical pain, insomnia, restlessness, crying, sudden reactions. Gradually, trust and security were built up.”* – Participant 7

The psychological and social burden emerged as a significant pathway through which homelessness shapes health outcomes. Participants repeatedly emphasised “no rest, no safety spaces, nowhere to go,” capturing how constant mobility, exhaustion and environmental exposure undermined homeless migrant s’ physical and mental health.

#### 3.4.3 Deterioration of mental and physical health under housing instability

This theme traces the cumulative and embodied effects of sustained structural vulnerability, showing how prolonged housing instability became inscribed in migrants' physical and mental health. Beyond direct medical risks, homelessness created deep psychological strain. Participants reported that homeless migrants had the need of being seen, respected and a place of their own. Not being recognized and being dependent on charity and others were among homeless migrants seen as unworthy and a threat to the persons dignity. Some EU migrants who struggled with the Norwegian system were proud and would rather sleep in the street than using the night shelter. Some undocumented migrants would rather stay in a tent in the forest than using the reception centres. Being homeless over time was to a certain degree experienced as an existential threat for undocumented migrants. The life in limbo for several years affected their mental health, ability to cope and they did not see the night shelters as an alternative. As one participant stated:

*“Homelessness over time in Norway significantly affects health – both physical and mental. Self-confidence and self-esteem.[…] It involves a kind of invisibility. You don't exist. You don't count. You are nobody. Just a homeless person that nobody cares about”* – Participant 1

The life on the street was hard and stressful and represented a health problem by itself. Norwegian climate is rough, especially during autumn and winter seasons with rain and temperatures that could drop to minus 20 degrees Celsius. Several noted persistent infections, untreated wounds, and deterioration of cardiovascular and respiratory symptoms due to harsh weather and lack of shelter. Across interviews, homelessness emerged as an active health risk rather than a contextual factor.

Homelessness interacted with migrants’ pre-existing vulnerabilities, such as precarious legal status, limited finances, language barriers, and untreated chronic disease to accelerate health decline. Participants described direct physical consequences of living outdoors, including cold exposure, poor hygiene conditions, and inability to rest. Homeless migrants got exhausted and needed a place to recover (Table 2).

Most of the homeless migrants at the long-term shelter used the Health Centre for undocumented migrants run by NGOs for primary healthcare. The staff characterized the users of the long-term shelter as with restricted rights and in a precarious situation which in combination challenged both the existing public and NGO service provision. The staff reported that homelessness and the precarious situation over time challenged health personnel ability to provide standard treatment for both physical and mental problems.

*“We see serious health problems in many people. It is about wear and tear that has developed into serious diagnoses – both mentally and physically. They are simply not caught well enough. The health center is doing a great job, it must be said, but they only manage to handle the tip of the iceberg. This applies especially to chronic ailments such as muscle and skeletal pain – back, legs, and so on – which no physiotherapist can fix, because it is in the body in a different way. These are mental challenges that have physical consequences.”* – Participant 6

Shielding was experienced by staff to have a positive effect on homeless migrants’ health. Having a place to resort to and sort things out helped them endure the life on the street or from sofa to sofa. People who were unpredictable and incoherent suddenly appeared calm and coherent after getting regular sleep and food. When having a place to stay over time the homeless migrants had the possibility to assess their own situation and options.

*“It's fascinating. We have a guest here. He's very unstable. A very kind man, but it's obvious that he has a diagnosis. We don't know what it is - a mix of ADHD maybe. He messes up a lot. Always comes with piles of papers. Talks very fast. He's always getting into trouble with employers and losing his jobs. It's very difficult to get an overview of his life. And then suddenly he disappeared. The last time he was with us, he came with a lot of letters from the police. It turned out that he had committed several offenses - riding an electric scooter at full speed in the street and at a red light and was stopped by the police. Lots, lots of stuff. And then suddenly he appeared again after three weeks - a completely different man. We were all completely fascinated. Fresh, calm, spoke normally, a normal expression on his face. Because before he had always looked very restless. I asked: "What happened? Where have you been?" He had been in prison - because of what he had done. And he had such a good time there. He got to sleep, he got to eat – because otherwise he lives on the streets. He was a completely different person. And it's fascinating.” –* Participant 8

The staff experienced the long-term shelter provided a better opportunity for them to assess what the homeless migrant needed of healthcare and what rights to health and social services he/she had. The staff needed to use time for homeless migrants to regain trust to others when being excluded from society. When the homeless migrant had a place to stay, the staff could reach them over time and facilitate trust in and access to healthcare. A part of this work was to reassure them so that they will accept help.

*“It's so complex. It's about scepticism about the system, the healthcare system, and authority in general. They bring with them established beliefs – sometimes myths – that are deeply rooted. The most important thing is to be present and listen: What is important to them? What bothers them? What are they afraid of? It's not just about information, but about safety and trust.”* – Participant 7

## 4 Discussion

In summary, our findings suggest that homelessness among migrants operates across multiple, interconnected levels and appears to play a central role in shaping health outcomes among migrants experiencing extreme and prolonged marginalisation. Homelessness thus emerges not merely as a background condition, but as an active mechanism through which vulnerability is reinforced and deepened, and over time, accumulated and embodied.

By combining the concepts of precarious and undeserving citizenship (Lafleur and Mescoli, 2018) with a structural vulnerability framework (Bourgois et al., 2017; Carruth et al., 2021), this study shows how migrants’ positioning within overlapping legal, administrative and socio-economic hierarchies constrains access to care, continuity of treatment and possibilities for recovery. These dynamics are particularly evident when access to healthcare and social support is mediated through humanitarian services. As demonstrated by Pursch et al. (2020), such arrangements are characterised by inherent power asymmetries, shifting responsibilities between state and NGOs, and disempowerment associated with conditional and temporary forms of aid.

### 4.1 Homelessness as a multilevel driver of inequality

Our findings align with and extend previous research demonstrating that homelessness and discrimination are closely associated with psychological distress, particularly among marginalized groups. Crucially, this relationship is bidirectional. Mental health problems, often rooted in prior trauma, exploitation or prolonged uncertainty can increase the risk of homelessness by undermining employment, income and social networks. At the same time, homelessness intensifies psychological distress through sustained exposure to insecurity, exhaustion and lack of control. Conceptually, we frame this dynamic as a form of structural vulnerability, in which precarious citizenship and welfare exclusion interact in ways consistent with cumulative and self-reinforcing health risks. Aligned with earlier literature (Keynejad et al., 2026) we thus identify a mutually reinforcing cycle in which housing instability contributes to ontological insecurity and stress, heightening vulnerability to violence and mental health problems, while deteriorating mental health further erodes the capacity to secure stable housing.

This cycle was evident across the empirical material. Migrants with chronic somatic illness, post-surgical conditions or mental health problems experienced disproportionate consequences of homelessness, as the absence of a safe place to rest, recover and adhere to treatment appeared to transform otherwise manageable conditions into prolonged or severe illness trajectories. While this resonates with earlier studies documenting disrupted care pathways among homeless populations (Hanlon et al., 2018; Luchenski et al., 2022), our analysis extends this literature by showing how migration status and welfare exclusion systematically intensify these disruptions. Restricted access to rehabilitation, follow-up care and social welfare appeared to actively reshape illness trajectories, illustrating how health deterioration among homeless migrants may be understood as situated at the intersection of clinical, administrative and structural domains.

### 4.2 Precarious citizenship, gender and capacity to respond

This study demonstrates how structural, relational and gendered vulnerability intersects. Gendered patterns were not uniform, but in our material, they appeared closely tied to legal status and the forms of precariousness migrants could be drawn into. Men described more often exploitation in the labour market and lack of access to interim support which could rapidly trigger homelessness following job loss. Women more often described exploitation in personal and informal relations, including coercive relationships, sexual services, and unpaid or underpaid care and domestic work. Together, these accounts indicate that precarious citizenship can be enacted through different arenas of exploitation, with consequences for safety, access to care and possibilities for recovery.

These findings contribute to earlier research on welfare bordering, discretionary exclusion and digital bordering in European welfare states (Bendixsen and Corrales-Øverlid, 2024; Czech-Havnerås and Schweyher, 2024; Lafleur and Mescoli, 2018; Misje, 2020). Holte & Misje (2024) shows how EU migrants positioned at the margins of the labour market are discursively constructed either as illegitimate workers or as social problems, such as “beggars” or “welfare seekers,” rather than as rights-bearing labour migrants. Our findings complicate this distinction by demonstrating that these categories are not clean-cut in practice. Migrants who enter Norway as workers may transition into homelessness following job loss, labour exploitation, wage theft or trafficking for forced labour, and subsequently face similar forms of material deprivation, stigma and unmet needs as those categorised as “beggars” in public discourse.

Together, these findings illustrate how health deterioration cannot be understood independently of the legal, administrative and discursive regimes that regulate access to welfare and define who is considered deserving of support. Migrants’ ability to mobilise resources, navigate systems and recover from illness was deeply shaped by their gendered position within these regimes, influencing both access to services and their recognition as legitimate patients and rights-holders.

### 4.3 Humanitarian responses and structural mitigation

Our findings underscore the limitations of humanitarian and project-based responses. While shelters and outreach services play an important bridging role, they are typically characterised by temporariness, conditionality and discretionary access. As documented across European contexts, including during the COVID-19 pandemic, temporary housing initiatives were often effective in meeting immediate needs but remained weakly integrated into welfare and housing systems and lacked long-term sustainability (Delvino and Spencer, 2019; Zufferey Cz et al., 2022). Once crisis measures were withdrawn, many of the structural conditions underlying homelessness and health vulnerability quickly re-emerged.

Evidence from more integrated and migrant-centred housing initiatives suggests that alternative models can mitigate this pattern. Projects combining stable housing with legal support, health follow-up and community integration have demonstrated potential to foster autonomy, dignity and longer-term inclusion when they move beyond emergency logic (Cassano et al., 2025; eClinicalMedicine, 2023; Johnson et al., 2025). Key elements include bottom-up design, recognition that temporary accommodation is insufficient, and an explicit focus on empowerment rather than risk management alone. Our findings support the relevance of such approaches and highlight the need to shift from humanitarian mitigation toward more stable, integrated and rights-based housing responses.

### 4.4 Strengths and limitations

This study has several limitations. The qualitative analysis is based on interviews with health and social care professionals rather than migrants themselves, which may underrepresent migrants’ own perspectives and agency. The study population is limited to migrants who accessed a long-term shelter and may therefore not capture the experiences of those who did not meet the shelter’s inclusion criteria and live entirely outside services, or those with more severe substance use or mental health problems.

Health outcomes were not clinically assessed but inferred from registry notes and staff accounts, limiting medical specificity. There is a potential of misclassification of reason for admission as only first author created the categories and that it is based on employee descriptions. Findings are situated within the Norwegian welfare context and may not be directly generalisable to other settings. Finally, the study period overlaps with the COVID-19 pandemic, during which temporary policy adaptations may have influenced access to housing and services.

While the heterogenous dataset limits comparability and precludes narrow typologies or causal inference, it also constitutes a strength by reflecting the complexity and fluidity of homelessness among migrants as it unfolds in practice.

Despite the limitations, the study provides rare insight into the multilevel health consequences of homelessness among migrants, a population largely absent from surveys and administrative data and identifies mechanisms of exclusion that are difficult to capture through quantitative approaches alone.

## 5 Conclusion

This study argues and demonstrates that homelessness among migrants is not merely a downstream consequence of social disadvantage, but a central mechanism through which structural forms of vulnerability are reinforced, intensified and embodied. Across individual, relational and structural levels, homelessness shapes exposure to illness, disrupts recovery and constrains access to care in ways that appear to restructure health trajectories. Health deterioration emerges not as an isolated clinical event, but as embodied within intersecting legal, administrative and socio-economic processes that regulate access to housing, healthcare and protection.

Addressing health inequities among homeless migrants therefore requires recognising homelessness as a structural issue rather than a temporary personal emergency. Without integrated and rights-based approaches that link housing stability, healthcare access and social protection, health and humanitarian systems risk institutionalising a form of managed marginality where suffering is alleviated, but structural vulnerability remains fundamentally intact.

## Declaration of generative AI and AI-assisted technologies in the manuscript preparation process

During the preparation of this manuscript, the authors used ChatGPT (OpenAI) to assist with language editing and improving clarity and structure of some parts of the text. After using this tool, the authors reviewed and edited all AI-assisted content and take full responsibility for the final content of the published article.

## Funding

This work was supported by the Norwegian Research Council, project, number 325188.

## CRediT authorship contribution statement

**Frode Eick:** Writing – original draft, Project administration, Methodology, Investigation, Formal analysis, Data curation, Conceptualization. **Synnøve Økland Jahnsen:** Writing – review & editing, Project administration, Methodology, Investigation, Funding acquisition, Formal analysis, Conceptualization.

## Declaration of competing interest

The authors declare no conflicts of interest.
